# Case Report: Diabetic ketoacidosis following EGFR-TKIs therapy via aggravated insulin resistance

**DOI:** 10.3389/fonc.2026.1862643

**Published:** 2026-07-31

**Authors:** Shengjie Wu, Li Jing, Yan Zhang

**Affiliations:** 1 Department of Pharmacy, Sir Run Run Shaw Hospital, School of Medicine, Zhejiang University, Hangzhou, China; 2 Department of Pharmacy, Nanjing First Hospital, Nanjing Medical University, Nanjing, China; 3 Diabetic Foot Multidisciplinary Treatment Center, Honghui Hospital of Xi’an Jiaotong University, Xi’an, China

**Keywords:** case report, diabetic ketoacidosis, EFGR-TKIs, insulin resistance, NSCLC

## Abstract

**Objectives:**

Epidermal growth factor receptor-tyrosine kinase inhibitors (EGFR-TKIs) are an established first-line treatment for non-small cell lung cancer (NSCLC) patients with sensitizing mutations because of their remarkable efficacy and favorable tolerability profiles. However, rare and severe adverse reactions during their application require attention. Cases of diabetic ketoacidosis (DKA) associated with EGFR-TKI therapy have seldom been reported and the underlying mechanism remains unclear.

**Case presentation:**

We present the case of a 64-year-old female patient with immune checkpoint inhibitor-induced diabetes mellitus (ICI-DM) on aumolertinib (a third-generation EGFR-TKI, 110 mg daily) for the treatment of NSCLC admitted to the hospital with frequent episodes of DKA. Owing to a recurrent DKA episode following a reduced dosage (55 mg daily), aumolertinib was suspended permanently. After full recovery from the DKA, the patient was switched to osimertinib (80 mg daily). Insulin resistance in the patient was assessed using a hyperinsulinemic-euglycemic clamp. She was maintained on standard-dose osimertinib and insulin therapy after discharge and remained asymptomatic during the outpatient follow-up.

**Conclusion:**

Owing to the protocol-defined strict inclusion and exclusion criteria, some potentially serious adverse reactions may not be as commonly observed in clinical trial settings. Aumolertinib-induced exacerbation of insulin resistance can precipitate a spectrum of hyperglycemia, ranging from mild elevations to severe DKA, which highlights the necessity of blood glucose monitoring throughout the treatment course. By assessing a patient’s tolerance profile for EGFR-TKIs-aggravated insulin resistance using the insulin clamp technique, clinicians can optimize antineoplastic regimens, such as switching from aumolertinib to osimertinib in this case.

## 1 Introduction

Immune checkpoint inhibitors (ICIs), such as anti-programmed cell death-1 (PD-1) antibodies, have revolutionized the treatment of advanced non-small cell lung cancer (NSCLC) (1). However, ICIs can trigger a spectrum of immune-related adverse events, among which ICI-induced diabetes mellitus (ICI-DM) is a rare but potentially life-threatening complication (2). ICI-DM typically presents with acute onset of hyperglycemia and diabetic ketoacidosis (DKA), often accompanied by a rapid and profound decline in beta-cell function, as evidenced by markedly reduced C-peptide levels (3, 4). Unlike classic type 1 diabetes, ICI-DM may occur in patients without prior history of diabetes and can develop at any time during or even after cessation of ICI therapy (3). The incidence of ICI-DM is estimated to be approximately 1%, and the number of recognized cases continues to rise with the widespread use of ICIs and improved clinical awareness (5).

Third-generation EGFR-TKIs have shown great efficacy and safety compared to platinum-based chemotherapy in several phase 3 trials and have been recommended as the standard first-line treatment for NSCLC patients harboring sensitizing EGFR mutations (exon 19 deletions, L858R point mutation, or T790M point mutation). TKIs have been reported to be associated with hyperglycemia through various proposed mechanisms (6). Although EGFR-TKIs-induced hyperglycemia is relatively uncommon (approximately 1% to 2.4% incidence, predominantly Grade 1) in randomized controlled trials, with the notable exception of rociletinib, which has an incidence as high as 55%, vigilance remains necessary in real-world clinical practice (7, 8).

Although the AENEAS trial safety data indicated a relatively low incidence (<1%) of severe (Grade ≥3) hyperglycemia with aumolertinib, vigilance against such serious adverse reactions remains warranted in clinical practice (9). Here we report a case of a patient with pre-existing ICI-DM who experienced recurrent DKA following initiation of aumolertinib, suggesting that EGFR-TKIs may exacerbate insulin resistance in this vulnerable population. Subsequent investigations confirmed that frequent episodes of DKA stemmed from aumolertinib-induced exacerbation of insulin resistance. After switching to osimertinib, the patient demonstrated good tolerance to therapy.

## 2 Case presentation

### 2.1 First diagnosed episode of DKA

**Figure 1:**
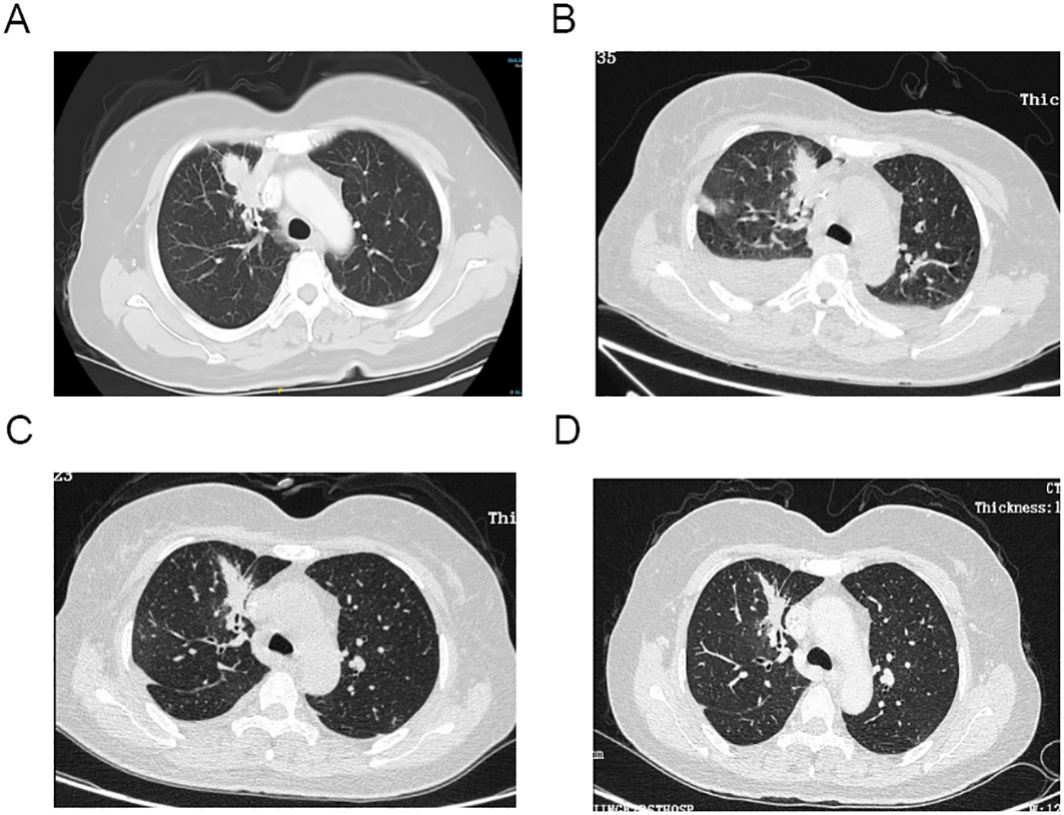
Chest CT images. **(A)** Former chest CT image. **(B-D)** Chest CT images of the first hospital admission, second hospital admission, and the outpatient follow-up, respectively.

A 64-year-old Han Chinese woman with a history of immune checkpoint inhibitor-induced diabetes mellitus (ICI-DM) for 2 years, diagnosed after developing DKA following pembrolizumab therapy presented at the emergency room with nausea and vomiting for 4 days, which worsened for 1 day. The patient’s medical history was significant for NSCLC (cTxNxM1, stage IV) and family history was unremarkable for diabetes or lung cancer. The patient’s psychosocial history was unremarkable either. The patient denied any alcohol or tobacco use. She was treated with aumolertinib monotherapy (Ameile, Hansoh, China) 110 mg daily for NSCLC with EGFR mutation (exon 20 T790M point mutation), and a basal-bolus insulin regimen for diabetes. The patient initiated aumolertinib at a dose of 110 mg once daily on May 24, 2022, for NSCLC and had been managed with a basal-bolus insulin regimen for ICI-DM since its diagnosis on November 2, 2020. The first episode of DKA occurred on May 31, 2022, approximately one week after starting aumolertinib. Her initial vital signs were as follows: blood pressure, 91/48 mmHg; heart rate, 137 beats/min; respiratory rate, 24/min; body temperature, 36.3°C; and blood oxygen saturation, 96%. Lung auscultation revealed diminished breath sounds in the right lung and moist crackles in both the lungs. Laboratory findings are listed in **Table 1**. Chest computed tomography (CT) revealed bilateral pleural effusion, ground-glass shadows in both lungs, and high-density patchy shadows in the upper lobe of the right lung (**Figure 1B**). An upper abdominal ultrasound scan indicated fatty liver, and echocardiography was unremarkable. The patient was diagnosed with DKA and initiated standard treatment regimens, including restoration of circulating volume, insulin therapy, and electrolyte replacement. In addition, treatment of any underlying precipitating event is also a central part in the management of DKA (10). Elevated plasma cytokines (such as IL-6 and IL-10), leukocytes, and neutrophils were significantly correlated with DKA (10), nevertheless pneumonia in this patient could not be completely ruled out after comparing with her former chest CT findings (**Figure 1A**). Therefore, ceftizoxime (2 g, twice daily) was administered.

**Table 1:** Laboratory findings of the patient*.

Items	First admission	Second admission	Third admission	Previous admission (2 years ago)
Admission random blood glucose level (mmol/L)	29.03	20.46	17.30	18.2
CBC: WBC (3.5-9.5×10^9^/L), NE (1.8-6.3×10^9^/L), Hb (115-150 g/L), RBC (3.8-5.1×10^12^/L), PLT (125-350×10^9^/L)	22.81↑, 20.00↑, 117, 3.75↓, 170	4.46, 2.62, 111↓, 3.56↓, 168	11.85↑, 9.27↑, 108↓, 3.49↓, 190	9.95↑, 7.93↑, 157↑, 4.99, 226
ABG: pH (7.35-7.45), PO_2_ (80-100 mmHg), PCO_2_ (35-45 mmHg), bicarbonate level (21.4-27.3 mmol/L), lactic acid level (0.5-1.7 mmol/L)	7.21↓, 160↑, 22↓, 8.8↓, 2.8↑	7.27↓, 91, 24↓, 13.60↓, 1.8↑	7.22↓, 200↑, 37, 15.10↓, 1.8↑	7.25↓, 108↑, 29↓, 12.70↓, 0.90
PCT (0-0.25 ng/ml), CRP (0-8 mg/L), IL-6 (0-7.0 pg/ml), SAA (0-10.0 mg/L)	11.63↑, 38.90↑, 127.4↑, 6.33	0.75↑, < 5, 21.00↑, < 5	4.69↑, 7.38, < 4, < 5	/
Serum ketone level (< 0.6 mmol/L)	5.2↑	5.7↑	6.1↑	5.7↑
KET (-)	/	/	+	/
HbA1c concentration (4.0-6.0%)	10.10%↑	/	8.60%↑	8.80%↑
ALT (7-40 U/L), AST (13-35 U/L), GGT (7-45 U/L), ALP (50-135 U/L)	5.10↓, 22.00, 8.10, 57.10	8.6, 27.40, 12.80, 70.20	4.5↓, 14.90, 11.50, 51.80	6.00↓, 13.00, 17.00, 61.00
TC (3.12-5.98 mmol/L), TG (0.44-1.70 mmol/L), HDL-C (1.00-1.90 mmol/L), LDL-C (0-3.10 mmol/L)	3.46, 1.21, 1.69, 1.41	5.35, 1.13, 2.12↑, 2.97	3.37, 1.00, 1.67, 1.38	5.00, 2.19↑, 1.35, 2.10
Fasting plasma glucose level (3.90-6.44 mmol/L), postprandial 2-hr plasma glucose level	10.23↑, 16.84	2.09↓,/	7.20↑,/	12.45↑,/
Fasting C-peptide level (0.78-5.19 ng/ml), postprandial 2-hr C-peptide level	<0.01↓, <0.01	/	<0.01↓,/	0.24↓, 0.27
Fasting insulin level (2.60-11.80 ng/ml), postprandial 2-hr insulin level	/	/	241.88↑,/	14.40↑, 40.10
Autoantibodies against islet: GADA (< 10.00 IU/ml), ICA (1.00 S/CO), IAA (< 1.00 COI)	1.97, 0.06, 0.20	/	/	4.30, 0.07, 0.07
FFA (100-450 μmol/L)	/	/	513↑, 28↓	/

*The hormone levels of thyroid, pituitary, parathyroid and adrenal were all within the normal limits, and serum creatine kinase, amylase and lipase levels were also unremarkable (data not shown). CBC, complete blood count, WBC, white blood cell count, NE, neutrophil count, Hb, hemoglobin, RBC, red blood cell count, PLT, platelet count, ABG, arterial blood gas, AG, anion gap, PO_2_, partial pressure of oxygen, PCO_2,_ partial pressure of carbon dioxide, PCT, procalcitonin, CRP, C-reactive protein, IL-6, interleukin- 6, SAA, serum amyloid A, KET, urinary ketone bodies, HbA1c, hemoglobin A1c, ALT, alanine transaminase, AST, aspartate transaminase, GGT, gamma-glutamyltransferase, ALP, alkaline phosphatase, TC, total cholesterol, TG, triglycerides, HDL-C, high-density lipoprotein cholesterol, LDL-C, low-density lipoprotein cholesterol, GADA, glutamic acid decarboxylase autoantibodies, ICA, islets cells autoantibodies, IAA, insulin autoantibodies, FFA, free fatty acid. Serum insulin level at the third admission was measured while the patient was receiving exogenous insulin infusion, thus does not reflect endogenous insulin secretion.

**Figure 2:**
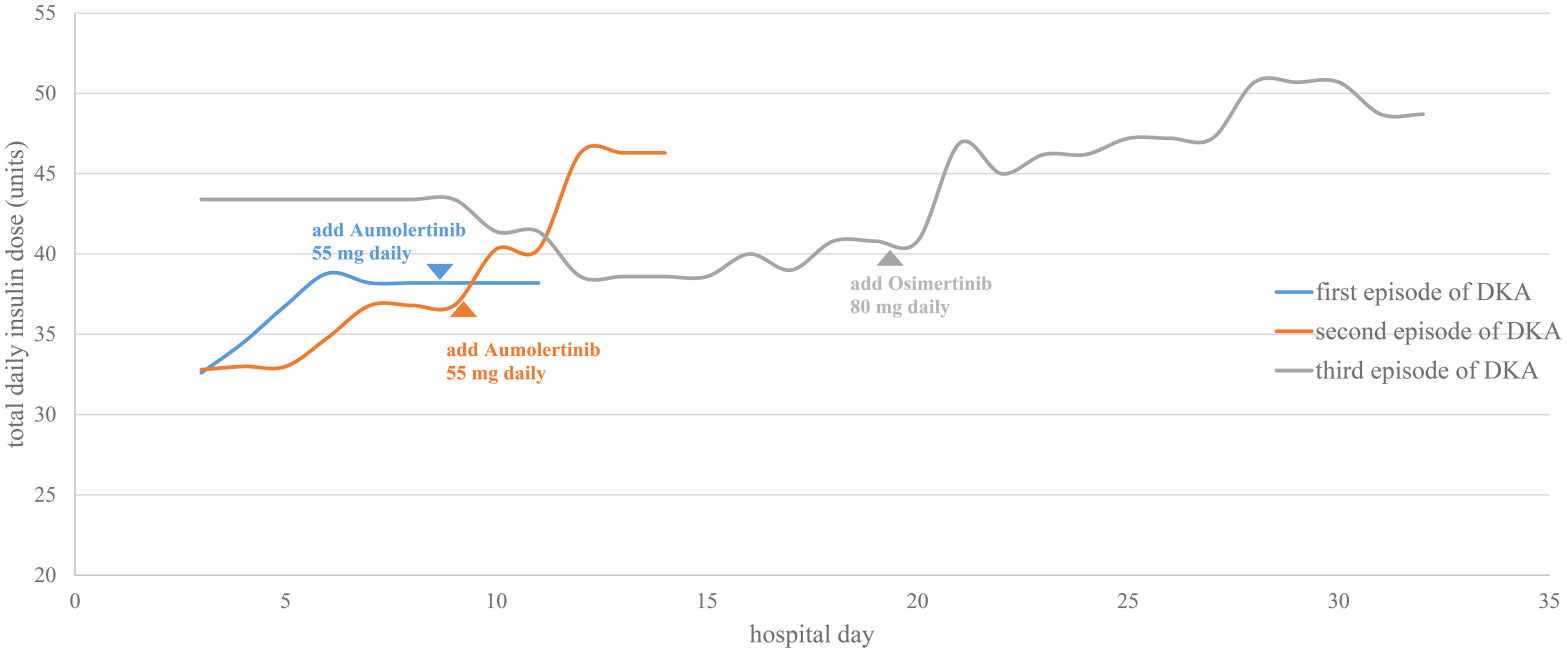
Daily insulin requirement. Total daily insulin dose of the first, second, and third hospital admission respectively.

Although no case of DKA or severe hyperglycemia has been observed with aumolertinib in clinical trials (9, 11, 12), osimertinib has been associated with dose-limiting hyperglycemia, which may necessitate dose reduction (13). We speculated that aumolertinib, as a novel, irreversible, third-generation EGFR-TKI, might also have the same adverse effect profiles. The causality assessment using the Naranjo algorithm yielded a possible association (total score of 4), resulting in the discontinuation of aumolertinib on the first day of admission (14). After resolution of hyperglycemia and acidosis, half of the recommended dose of aumolertinib (55 mg daily) was initiated, and her blood glucose levels were closely monitored for another two days (**Figure 2**, blue line). On the eleventh day of admission, her blood glucose level achieved glycemic targets without any hypoglycemic events, and she was discharged with insulin pump therapy. Before admission, the patient was using a basal-bolus insulin regimen, the specific details of which are unknown. During the hospitalization, we also noted the changes in the daily insulin requirements: At the first admission, the initial intravenous insulin infusion rate was 5 U/h, and it was subsequently adjusted to continuous subcutaneous insulin infusion (CSII), with the maximum daily dose reaching 38.2 units; at the second admission, the maximum daily dose of CSII was 43.6 units; at the third admission, the maximum daily dose of CSII was 50.7 units.

### 2.2 Second occurrence of DKA

Nine days later, she was readmitted to the emergency room with DKA (random glucose 20.46 mmol/L, pH 7.27, serum ketones 5.7 mmol/L) accompanied by 2 days of nausea and vomiting. On presentation, the patient was afebrile with normal vital signs. Laboratory findings are shown in **Table 1**. A routine chest CT scan demonstrated that the bilateral pleural effusion and high-density patchy shadows in the upper lobe of the right lung were partly absorbed (**Figure 1C**). After the first discharge, the patient resumed taking aumolertinib at 55 mg once daily for 9 days. Due to good blood sugar control and after consultation with the oncology attending physician who stated that there were no relevant ADR reports in the current clinical study, it was recommended to resume the dosage at 110 mg once daily. However, DKA occurred 6 days after the dosage increase. According to the patient herself, her blood glucose levels had gradually increased ever since, fluctuating between 15.6 mmol/L to 26.3 mmol/L (Grade 3 hyperglycemia). Therefore, we initiated the same treatment regimen. Reassessment using the Naranjo Scale yielded an elevated score of 11 (definite association). Hence, the dose of aumolertinib was reduced to 55 mg daily (**Figure 2**, orange line). On hospital day 12​, the patient was discharged on insulin pump therapy.

### 2.3 Subsequent episode of DKA

Unfortunately, 26 days later, for the third time, she presented with DKA (also without apparent causes) and a 1-day history of nausea and vomiting. Her vital signs were unremarkable except for a mild elevation of heart rate to 110 beats/min. Laboratory findings are shown in **Table 1**. Owing to the recurrence of DKA even after dose reduction, aumolertinib was suspended permanently. To identify an alternative antitumor treatment regimen, we reviewed her former NSCLC chemotherapy regimens.

Two years after the initial treatment with icotinib, the patient received three cycles of pembrolizumab, bevacizumab and docetaxel owing to tumor progression. However, one and a half months later, she presented with polydipsia, polyuria and emaciation. The patient was diagnosed with DKA, and the fourth cycle of chemotherapy treatment was discontinued because of the severe adverse reaction of DKA associated with pembrolizumab. She denied a history of diabetes, and her fasting plasma glucose levels were 6–7 mmol/L prior to the ​​initiation of​​ pembrolizumab. The patient did not receive any further systemic anticancer therapy for approximately 18 months before initiation of aumolertinib. Based on her symptoms, past medical history, medication history and laboratory results (**Table 1**), a diagnosis of ICI-DM should be considered.

TKIs have been reported to induce hyperglycemia by increasing insulin resistance and reducing beta-cell function (7). After obtaining informed consent, we assessed aumolertinib-associated insulin resistance using the hyperinsulinemic-euglycemic clamp (HEC) following the resolution of DKA in this patient (15). On hospital day 11(aumolertinib withdrawn for 7 days), the M-value (glucose disposal rate) was 2.6 mg/kg·min (normal range: 4.7-8.7 mg/kg·min) (16, 17), and ten days later (aumolertinib withdrawn for 17 days), the M-value was rose to 3.6 mg/kg·min. As for the alternative treatment options for NSCLC, it seemed like rechallenging with other third-generation EGFR-TKIs such as osimertinib (Tagrisso, AstraZeneca, Sweden), which might also be risky. After fully informing the patient of the potential severe adverse reactions to osimertinib, she agreed to rechallenge with osimertinib. On hospital day 19, osimertinib (80 mg daily) was initiated, and her blood glucose level was closely monitored (**Figure 2**, gray line). On hospital day 31 (osimertinib added for 12 days), the HEC test was performed for the third time, and the M-value decreased to 3.2 mg/kg·min. Although metformin, dipeptidyl-4 inhibitors, and glucagon-like peptide-1 receptor agonists have been recommended for improving insulin resistance, they are not recommended for the treatment of ICI-DM. The patient refused to take any of these medicines. Therefore, the patient was treated with a basal-bolus insulin regimen.

At the two-month outpatient follow-up, the patient was maintained on​ a regular regimen of osimertinib and insulin pump therapy, and remained asymptomatic. After switching to osimertinib, the patient experienced no recurrent DKA episodes, and her glycemic control improved compared to the period on aumolertinib, although blood glucose levels occasionally exceeded the target range (**Table 2**), and serum ketone levels were normal (< 0.6 mmol/L). Repeated chest CT indicated the continued response to osimertinib (**Figure 1D**). The patient was pleased with the therapeutic effect achieved by the current treatment regimen. We recommended another HEC test, but the patient refused. We suggest that the patient monitor blood glucose and ketones levels closely, get a follow-up check (such as chest CT scan) regularly, and contact us immediately if necessary. Moreover, we will continue to follow-up the patient in the future.

**Table 2:** Self-monitoring of blood glucose records for the 10-day period preceding follow-up (mmol/L).

Day	Fasting blood glucose	Preprandial blood glucose (lunch)	Preprandial blood glucose (dinner)
1	12.9	10.5	8.5
2	9.3	4.3	7.5
3	6.0	11.2	12.0
4	11.3	9.3	6.7
5	8.8	3.9	4.2
6	7.3	13.3	3.8
7	8.8	5.5	9.6
8	9.5	6.9	4.4
9	11.7	13.6	11.5
10	10.7	12.9	10.7

## 3 Discussion

Immune checkpoint inhibitors (ICIs), a class of monoclonal antibodies targeting cytotoxic T lymphocyte antigen-4 (CTLA-4) or programmed cell death-1 and its ligand (PD-1/PD-L1), have been shown to improve overall survival in numerous cancer types. However, ICIs can also cause a wide-array of immune-related adverse events that can range from mild to life-threaenting. ICI-DM occurs in up to 1% of patients treated with ICIs, and the number has been increasing owing to the early recognition and diagnosis (18). Most patients develop ICI-DM while on therapy, and a large proportion present with DKA with relatively mild elevation of hemoglobin A1c (HbA1c), indicating an abrupt decline in insulin secretory function (18). In this case, the patient developed DKA following treatment with pembrolizumab, accompanied by a moderately elevated HbA1c and a dramatic decline in both fasting and 2-hour postprandial C-peptide levels (**Table 1**). These findings conclusively confirm the diagnosis of ICI-DM. Previous history of diabetes though she denied, based on her elevated fasting plasma glucose (6–7 mmol/L) before experiencing pembrolizumab and elevated HbA1c (8.8%), ICIs associated exacerbation of unrecognized underlying type 2 diabetes mellitus may also need to be considered (19).

Although ICI-DM often clinically resembles type 1 diabetes mellitus, the extent and mechanisms of insulin resistance in this condition are not well defined. Jehl et al. described a case of diabetes with severe insulin resistance that occurred during the course of anti-PD-1 immune therapy (20). Emerging evidence suggests that insulin resistance represents a distinct subtype within the clinical spectrum of ICI-DM (21). In the case, the patient also presented with insulin resistance (described in detail below), however, it remains unclear whether the observed insulin resistance is specifically related to ICI-DM itself or to the unmasking of a pre-existing, subclinical type 2 diabetes mellitus.

EGFR mutations in patients with NSCLC confer sensitivity to TKIs such as erlotinib, gefitinib, and afatinib. However, most patients develop resistance to prior EGFR-TKIs after 8–16 months, and 60-70% of patients harbor an acquired resistance mutation of T790M (22). Up to date, EGFR-TKIs targeting T790M have been developed to overcome resistance with favorable progression-free survival (PFS). Third-generation EGFR-TKIs, including osimertinib and aumolertinib, have been approved by China’s National Medical Products Administration for targeting the T790M mutation. Might not be very common though, EGFR-TKIs have been reported to be related with hyperglycemia, requiring dose reduction, oral antihyperglycemic, without discontinuation of therapy. We herein describe the first reported case of DKA induced by aumolertinib in a patient with ICI-DM, which was mediated by the exacerbation of insulin resistance.

The hyperinsulinemic-euglycemic clamp technique, first described by DeFronzo et al. (15), is regarded as the gold standard for assessing insulin sensitivity in both basal and insulin-stimulated states under various physiological conditions. We then assessed insulin action in the patient using an insulin clamp. Following the withdrawal of aumolertinib, the M-value rose from 2.6 mg/kg·min (at 7 days) to 3.6 mg/kg·min (at 17 days). This increase indicated a recovery of insulin sensitivity, thereby supporting the role of aumolertinib in mediating insulin resistance. Intriguingly, the M-value remained below 4.93 mg/kg·min even 17 days after aumolertinib withdrawal, which is the threshold indicating insulin resistance in normal weight, normal glucose tolerance individuals of the Chinese population (23). This suggests that ICI-DM itself may be complicated by underlying insulin resistance. When osimertinib was added for 12 days, the M-value decreased to 3.2 mg/kg·min, indicating mild exacerbation of insulin resistance. However, in studies of insulin-resistant individuals at euglycemia, much of the measured glucose uptake may be related to the effect of glucose itself on its own utilization, rather than to insulin-stimulated uptake, and this insulin-independent effect, which may differ depending on the metabolic and pathophysiological context, has the potential to confound the interpretation of the M value (16). More importantly, this is merely a case report with inherent bias, and large-scale studies are still needed in the future to assess the association between EGFR-TKIs and insulin resistance.

The molecular mechanisms underlying EGFR-TKI-associated insulin resistance remain unknown. Insulin resistance is related to hepatic, peripheral and adipose tissues, and EGFR is expressed in many tissues and cells, including adipocytes, skeletal muscle, and hepatocytes. However, selective deletion of EGFR in insulin-sensitive tissues failed to alter insulin resistance (24). Meanwhile, M460 and M502, two major metabolites of rociletinib, have been reported to exhibit activity against insulin growth factor-1 receptor and insulin receptor, which likely resulted in rociletinib-induced hyperglycemia (25). Whether aumolertinib or osimertinib-associated insulin resistance results from their antagonistic activity towards insulin growth factor-1 receptor and insulin receptor needs to be further studied.

## 4 Conclusion

We describe a unique case of immune checkpoint inhibitors-induced diabetes mellitus (ICI-DM) manifesting as recurrent diabetic ketoacidosis (DKA) in a patient treated with aumolertinib. Certain therapeutic agents, including aumolertinib and osimertinib, might potentiate insulin resistance to varying degrees. Therefore, when prescribing EGFR-TKIs to such patients, caution should be paid to blood glucose and ketone levels in cases of severe hyperglycemia or DKA. Based on our experience, we propose the following management strategies for EGFR-TKIs-induced hyperglycemia: 1. Withhold EGFR-TKIs until blood glucose levels return to the target range and symptoms resolve. Upon re-initiation, dose reduction may be preferred, accompanied by close glucose monitoring. 2. In cases of recurrent DKA or persistent severe hyperglycemia despite dose reduction, switching to an alternative EGFR-TKI with a more favorable metabolic profile should be considered. Whenever feasible, guiding this decision by assessing insulin resistance and secretion capacity is highly recommended.

## Data Availability

The original contributions presented in the study are included in the article/supplementary material. Further inquiries can be directed to the corresponding author.
